# Supplementary material for: Bacteriophage specificity is impacted by interactions between bacteria
Source: mSystems. 2024 Feb 20;9(3):e01177-23. doi: 10.1128/msystems.01177-23 (PMC11237722; doi:10.1128/msystems.01177-23)
Supplement: File S1 — Supplemental fixed point analysis. [file msystems.01177-23-s0001.pdf]

General equations and fixed points for the 4-species system

Supplementary Material for the manuscript “Bacteriophage diet breadth is impacted by interactions between bacteria.” This *Mathematica* file contains analytical calculations used to derive fixed point concentrations and corresponding eigenvalues. Queries may be addressed to Ave T. Bisesi at bis-es004@umn.edu.

Define the four equations, where X is *E. coli*, Y is *S. enterica*, g is the generalist phage, and s is the specialist phage. Throughout, this notebook will use this notation to distinguish between the four species. Once equations are defined, all equations are set equal to 0 and the system is simplified with regard to X, Y, g and s.

In[1]:=
```
Clear[mu1, mu2, b1, b2, a1, a2, k1, k2, z1, z2, R, v1, v2, δ]
xRHS = mu1 * X * ((a1 * Y) / (a1 * Y) + k1) * (R - X - b1 * Y) - z1 * g * X - δ * X;
yRHS = mu2 * Y * ((a2 * X) / (a2 * X) + k2) * (R - Y - b2 * X) - z2 * s * Y - z1 * g * Y - δ * Y;
gRHS = v1 * z1 * g * Y + v1 * z1 * g * X - δ * g;
sRHS = v2 * z2 * s * Y - δ * s;
fps =
  Simplify[Solve[{xRHS == 0, yRHS == 0, gRHS == 0, sRHS == 0}, {X, Y, g, s}], Reals];
```

Get all fixed points of the system of equations from the list of fixed points fps, of which there are 10. Each fixed point is labeled with the species that are present. For example, fpsXY is the fixed point at which only *E. coli* and *S. enterica* are present. For brevity, only a single example fixed point solution (fpsXY) is printed to demonstrate .

In[7]:=
```
fpsXY = FullSimplify[(fps⟦1⟧ // Normal)];
fpsY = FullSimplify[(fps⟦2⟧ // Normal)];
fpsYgen = FullSimplify[(fps⟦3⟧ // Normal)];
fpsYsp = FullSimplify[(fps⟦4⟧ // Normal)];
fpsXYgen = FullSimplify[(fps⟦5⟧ // Normal)];
fpsX = FullSimplify[(fps⟦6⟧ // Normal)];
fpsnone = FullSimplify[(fps⟦7⟧ // Normal)];
fpsXgen = FullSimplify[(fps⟦8⟧ // Normal)];
fpsXYsp = FullSimplify[(fps⟦9⟧ // Normal)];
fpsXYspgen = FullSimplify[(fps⟦10⟧ // Normal)];
fpsXY
```

Out[17]=

$$\left\{X \to \frac{(1+k2)\ mu2\ \delta + (1+k1)\ mu1\ ((-1+b1)\ (1+k2)\ mu2\ R - b1\ \delta)}{(-1+b1\ b2)\ (1+k1)\ (1+k2)\ mu1\ mu2},\right.$$
$$\left. Y \to \frac{-b2\ (1+k2)\ mu2\ \delta + (1+k1)\ mu1\ ((-1+b2)\ (1+k2)\ mu2\ R + \delta)}{(-1+b1\ b2)\ (1+k1)\ (1+k2)\ mu1\ mu2},\ g \to 0,\ s \to 0\right\}$$

We are interested in the stability of each fixed point. To determine this, we will first define the Jacobian matrix, and then create the Jacobian matrices evaluated on each fixed point. As a result, J2 will represent the Jacobian evaluated on the fixed point where only *S. enterica is* present. J2 is printed to demon-

strate the matrix form.

In[18]:=
```
J = {{D[xRHS, X], D[xRHS, Y], D[xRHS, g], D[xRHS, s]}, {D[yRHS, X], D[yRHS, Y],
     D[yRHS, g], D[yRHS, s]}, {D[gRHS, X], D[gRHS, Y], D[gRHS, g], D[gRHS, s]},
    {D[sRHS, X], D[sRHS, Y], D[sRHS, g], D[sRHS, s]}} // FullSimplify;
J1 = J /. fpsXY;
J2 = J /. fpsY;
J3 = J /. fpsYgen;
J4 = J /. fpsYsp;
J5 = J /. fpsXYgen;
J6 = J /. fpsX;
J7 = J /. fpsnone;
J8 = J /. fpsXgen;
J9 = J /. fpsXYsp;
J10 = J /. fpsXYspgen;
Style[MatrixForm[J2], 8]
```

Out[29]=

$$\begin{pmatrix} -\delta + (1+\text{k1})\,\text{mu1}\left(R - \text{b1}\left(R - \frac{\delta}{\text{mu2}+\text{k2 mu2}}\right)\right) & 0 & 0 & 0 \\ -\text{b2}\,(1+\text{k2})\,\text{mu2}\left(R - \frac{\delta}{\text{mu2}+\text{k2 mu2}}\right) & -\delta + (1+\text{k2})\,\text{mu2}\left(R - 2\left(R - \frac{\delta}{\text{mu2}+\text{k2 mu2}}\right)\right) & -\text{z1}\left(R - \frac{\delta}{\text{mu2}+\text{k2 mu2}}\right) & -\text{z2}\left(R - \frac{\delta}{\text{mu2}+\text{k2 mu2}}\right) \\ 0 & 0 & -\delta + \text{v1 z1}\left(R - \frac{\delta}{\text{mu2}+\text{k2 mu2}}\right) & 0 \\ 0 & 0 & 0 & -\delta + \text{v2 z2}\left(R - \frac{\delta}{\text{mu2}+\text{k2 mu2}}\right) \end{pmatrix}$$

To understand the linear stability of each fixed point, we will find the eigenvalues of each matrix by setting the determinant of the matrix (Jacobian - λ*I) equal to 0. Eigens2 - the eigenvalues of the Jacobian evaluated on the fixed point with only *S. enterica* present - is printed to demonstrate the form.

In[30]:=
```
Clear[mu1, mu2, b1, b2, a1, a2, k1, k2, z1, z2, R, v1, v2, δ]
eigens1 = Solve[Det[J1 - λ * IdentityMatrix[4]] == 0, λ];
eigens2 = Solve[Det[J2 - λ * IdentityMatrix[4]] == 0, λ] ;
eigens3 = Solve[Det[J3 - λ * IdentityMatrix[4]] == 0, λ] ;
eigens4 = Solve[Det[J4 - λ * IdentityMatrix[4]] == 0, λ] ;
eigens5 = Solve[Det[J5 - λ * IdentityMatrix[4]] == 0, λ] ;
eigens6 = Solve[Det[J6 - λ * IdentityMatrix[4]] == 0, λ] ;
eigens7 = Solve[Det[J7 - λ * IdentityMatrix[4]] == 0, λ] ;
eigens8 = Solve[Det[J8 - λ * IdentityMatrix[4]] == 0, λ] ;
eigens9 = Solve[Det[J9 - λ * IdentityMatrix[4]] == 0, λ];
eigens10 = Solve[Det[J10 - λ * IdentityMatrix[4]] == 0, λ];
eigens2
```

Out[41]=

$$\left\{\{\lambda \to -\text{mu2 R} - \text{k2 mu2 R} + \delta\},\ \left\{\lambda \to \frac{1}{(1+\text{k2})\ \text{mu2}}\right.\right.$$
$$(\text{mu1 mu2 R} - \text{b1 mu1 mu2 R} + \text{k1 mu1 mu2 R} - \text{b1 k1 mu1 mu2 R} + \text{k2 mu1 mu2 R} - \text{b1 k2 mu1 mu2 R} +$$
$$\left.\text{k1 k2 mu1 mu2 R} - \text{b1 k1 k2 mu1 mu2 R} + \text{b1 mu1 }\delta + \text{b1 k1 mu1 }\delta - \text{mu2 }\delta - \text{k2 mu2 }\delta)\right\},$$
$$\left\{\lambda \to \frac{\text{mu2 R v1 z1} + \text{k2 mu2 R v1 z1} - \text{mu2 }\delta - \text{k2 mu2 }\delta - \text{v1 z1 }\delta}{(1+\text{k2})\ \text{mu2}}\right\},$$
$$\left.\left\{\lambda \to \frac{\text{mu2 R v2 z2} + \text{k2 mu2 R v2 z2} - \text{mu2 }\delta - \text{k2 mu2 }\delta - \text{v2 z2 }\delta}{(1+\text{k2})\ \text{mu2}}\right\}\right\}$$

Cooperative parameters

To understand the stability of these fixed points when bacteria cooperate, mutualistic parameters consistent with Table 1 are included. In this case, beta values are set to 0, k values are set to 1, mu values are set to 0.5, R is set to 1, and the specialist’s burst size and attachment rate are initially equal with that of the generalist. For the purposes of fixed point analysis, the generalist always has the same burst size and attachment rate on both *E. coli* and *S. enterica*. A cost of generalism is imposed by increasing the attachment rate or the burst size of the specialist.

For a point to be stable, all eigenvalues must be less than 0. For simplicity, we will ignore fixed points where some or all values are greater than zero, indicating that the point is unstable or a saddle. For this reason, we will identify points of interest as those where the largest of the four eigenvalues is NOT greater than zero. These are points that, by definition, must be stable.

First, we will test our default parameters. Here, the only stable fixed point is the point where the two bacterial species and the generalist are present (eigens5). The code below will print out the number corresponding to any stable fixed points.

In[42]:=
```
Clear[mu1, mu2, b1, b2, a1, a2, k1, k2, z1, z2, R, v1, v2, δ]
mu1 = 0.5; mu2 = 0.5;
a1 = 1; a2 = 1;
k1 = 1;
k2 = 1;
R = 1;
δ = 3 / 100; b1 = 0; b2 = 0; v1 = 20; z1 = 1 / 1000;  z2 = 1 / 1000; v2 = 20;
list = {Max[Re[Flatten[{λ} /. eigens1]]],
   Max[Re[Flatten[{λ} /. eigens2]]], Max[Re[Flatten[{λ} /. eigens3]]],
   Max[Re[Flatten[{λ} /. eigens4]]],
   Max[Re[Flatten[{λ} /. eigens5]]], Max[Re[Flatten[{λ} /. eigens6]]],
   Max[Re[Flatten[{λ} /. eigens7]]], Max[Re[Flatten[{λ} /. eigens8]]],
   Max[Re[Flatten[{λ} /. eigens9]]], Max[Re[Flatten[{λ} /. eigens10]]]};
Position[list, _?(# < 0 &)]
```

Out[45]=
```
{{5}}
```

We will then test the stability of the fixed points when the specialist’s burst size is twice that of the generalist (v2 = 40). In this case, only eigens10 is stable, the point at which all four species are present.

In[ ]:=
```
Clear[mu1, mu2, b1, b2, a1, a2, k1, k2, z1, z2, R, v1, v2, δ]
mu1 = 0.5; mu2 = 0.5;
a1 = 1; a2 = 1;
k1 = 1;
k2 = 1;
R = 1;
δ = 3 / 100; b1 = 0; b2 = 0; v1 = 20; z1 = 1 / 1000;  z2 = 1 / 1000; v2 = 40;
list = {Max[Re[Flatten[{λ} /. eigens1]]],
   Max[Re[Flatten[{λ} /. eigens2]]], Max[Re[Flatten[{λ} /. eigens3]]],
   Max[Re[Flatten[{λ} /. eigens4]]],
   Max[Re[Flatten[{λ} /. eigens5]]], Max[Re[Flatten[{λ} /. eigens6]]],
   Max[Re[Flatten[{λ} /. eigens7]]], Max[Re[Flatten[{λ} /. eigens8]]],
   Max[Re[Flatten[{λ} /. eigens9]]], Max[Re[Flatten[{λ} /. eigens10]]]};
Position[list, _?(# < 0 &)]
```

Out[ ]=
```
{{10}}
```

We find that if the specialist’s burst size surpasses 2.83 that of the generalist (v2 > 57), then the only stable fixed point is eigens9, that of the two bacterial species plus the specialist.

In[ ]:=
```
Clear[mu1, mu2, b1, b2, a1, a2, k1, k2, z1, z2, R, v1, v2, δ]
mu1 = 0.5; mu2 = 0.5;
a1 = 1; a2 = 1;
k1 = 1;
k2 = 1;
R = 1;
δ = 3 / 100; b1 = 0; b2 = 0; v1 = 20; z1 = 1 / 1000; z2 = 1 / 1000; v2 = 57;
list = {Max[Re[Flatten[{λ} /. eigens1]]],
   Max[Re[Flatten[{λ} /. eigens2]]], Max[Re[Flatten[{λ} /. eigens3]]],
   Max[Re[Flatten[{λ} /. eigens4]]],
   Max[Re[Flatten[{λ} /. eigens5]]], Max[Re[Flatten[{λ} /. eigens6]]],
   Max[Re[Flatten[{λ} /. eigens7]]], Max[Re[Flatten[{λ} /. eigens8]]],
   Max[Re[Flatten[{λ} /. eigens9]]], Max[Re[Flatten[{λ} /. eigens10]]]};
Position[list, _?(# < 0 &)]
```

Out[ ]=
```
{{9}}
```

This principle holds for attachment rate, as well, if it is changed independently of burst size to impose a cost of generalism.

Importantly, if the product of the specialist's attachment rate and burst size is between 2 and 2.83 times that of the generalist, then the four species fixed point is the only stable point. We test that here, with v2 = 35 and z2 = 1.4 / 1000 such that the specialist's infectivity (attachment rate * burst size) is 2.45 times that of the generalist.

In[ ]:=
```
Clear[mu1, mu2, b1, b2, a1, a2, k1, k2, z1, z2, R, v1, v2, δ]
mu1 = 0.5; mu2 = 0.5;
a1 = 1; a2 = 1;
k1 = 1;
k2 = 1;
R = 1;
δ = 3 / 100; b1 = 0; b2 = 0; v1 = 20; z1 = 1 / 1000; z2 = 1.4 / 1000; v2 = 35;
list = {Max[Re[Flatten[{λ} /. eigens1]]],
   Max[Re[Flatten[{λ} /. eigens2]]], Max[Re[Flatten[{λ} /. eigens3]]],
   Max[Re[Flatten[{λ} /. eigens4]]],
   Max[Re[Flatten[{λ} /. eigens5]]], Max[Re[Flatten[{λ} /. eigens6]]],
   Max[Re[Flatten[{λ} /. eigens7]]], Max[Re[Flatten[{λ} /. eigens8]]],
   Max[Re[Flatten[{λ} /. eigens9]]], Max[Re[Flatten[{λ} /. eigens10]]]};
Position[list, _?(# < 0 &)]
```

Out[ ]=
```
{{10}}
```

Other patterns are not demonstrated here, but are trivial to investigate using the code provided above. These observations are that:

1) As the burst size or attachment rate of the specialist continues to increase relative to the generalist, the only stable fixed point remains the two bacterial species point with the specialist (eigens9).

2) The lower that the generalist’s infectivity (2* attachment rate * burst size, given that it can infect two species) is relative to delta (intrinsic mortality), the more likely it is that there is no region where the four species fixed point is stable (eigens10), instead immediately switching to eigens9 (bacteria plus specialist).

3) These trends shown here are largely invariant to growth rates and alpha values; increasing the benefit or growth rate of *E. coli* simply shifts these observed ranges to the right (see Figure 3C in the manuscript, which visualizes this).

Competitive parameters

We will perform many of the same calculations to understand the stability of these fixed points when bacteria are competing. However, we will instead use the competitive parameters consistent with Table 1. In this case, beta values are set to approximate 1, k values are set to 0, mu values are set to 0.5, R is set to 2, alpha values are set to 1, and the specialist’s burst size and attachment rate are initially equal with that of the generalist. As before, for the purposes of fixed point analysis, the generalist always has the same burst size and attachment rate on both *E. coli* and *S. enterica*. A cost of generalism is imposed by increasing the attachment rate or the burst size of the specialist.

First, we will test our default parameters. Here, the only stable fixed point is the point where the two bacterial species and the generalist are present (eigens5). This is only true if beta values approximate 1; if they are exactly 1, there are no stable fixed points.

In[ ]:=
```
Clear[mu1, mu2, b1, b2, a1, a2, k1, k2, z1, z2, R, v1, v2, δ]
mu1 = 0.5; mu2 = 0.5;
a1 = 1; a2 = 1;
k1 = 0;
k2 = 0;
R = 2;
δ = 3 / 100; b1 = 0.9999; b2 = 0.9999;
v1 = 20; z1 = 1 / 1000;  z2 = 1 / 1000; v2 = 20;
list = {Max[Re[Flatten[{λ} /. eigens1]]],
   Max[Re[Flatten[{λ} /. eigens2]]], Max[Re[Flatten[{λ} /. eigens3]]],
   Max[Re[Flatten[{λ} /. eigens4]]],
   Max[Re[Flatten[{λ} /. eigens5]]], Max[Re[Flatten[{λ} /. eigens6]]],
   Max[Re[Flatten[{λ} /. eigens7]]], Max[Re[Flatten[{λ} /. eigens8]]],
   Max[Re[Flatten[{λ} /. eigens9]]], Max[Re[Flatten[{λ} /. eigens10]]]};
Position[list, _?(# < 0 &)]
```

Out[ ]=
```
{{5}}
```

We will then test the stability of the fixed points when the specialist's burst size is twice that of the generalist (v2 = 40). There is no stable fixed point in this case. Note that this is only true if v2 is exactly 40, i.e. the specialist's burst size is exactly twice that of the generalist.

In[ ]:=
```
Clear[mu1, mu2, b1, b2, a1, a2, k1, k2, z1, z2, R, v1, v2, δ]
mu1 = 0.5; mu2 = 0.5;
a1 = 1; a2 = 1;
k1 = 0;
k2 = 0;
R = 2;
δ = 3 / 100; b1 = 0.9999; b2 = 0.9999;
v1 = 20; z1 = 1 / 1000;  z2 = 1 / 1000; v2 = 40;
list = {Max[Re[Flatten[{λ} /. eigens1]]],
   Max[Re[Flatten[{λ} /. eigens2]]], Max[Re[Flatten[{λ} /. eigens3]]],
   Max[Re[Flatten[{λ} /. eigens4]]],
   Max[Re[Flatten[{λ} /. eigens5]]], Max[Re[Flatten[{λ} /. eigens6]]],
   Max[Re[Flatten[{λ} /. eigens7]]], Max[Re[Flatten[{λ} /. eigens8]]],
   Max[Re[Flatten[{λ} /. eigens9]]], Max[Re[Flatten[{λ} /. eigens10]]]};
Position[list, _? (# < 0 &)]
```

Out[ ]=
```
{}
```

We will then test the stability of the fixed points when the specialist's burst size is greater than twice that of the generalist (v2 = 60). In this case, only eigens10 is stable, the point at which all four species are present. Again, this is only true if beta values approximate, rather than exactly equal, 1. This result continues to hold true as the specialist's burst size increases many times over that of the generalist's.

In[ ]:=
```
Clear[mu1, mu2, b1, b2, a1, a2, k1, k2, z1, z2, R, v1, v2, δ]
mu1 = 0.5; mu2 = 0.5;
a1 = 1; a2 = 1;
k1 = 0;
k2 = 0;
R = 2;
δ = 3 / 100; b1 = 0.9999; b2 = 0.9999;
v1 = 20; z1 = 1 / 1000;  z2 = 1 / 1000; v2 = 60;
list = {Max[Re[Flatten[{λ} /. eigens1]]],
   Max[Re[Flatten[{λ} /. eigens2]]], Max[Re[Flatten[{λ} /. eigens3]]],
   Max[Re[Flatten[{λ} /. eigens4]]],
   Max[Re[Flatten[{λ} /. eigens5]]], Max[Re[Flatten[{λ} /. eigens6]]],
   Max[Re[Flatten[{λ} /. eigens7]]], Max[Re[Flatten[{λ} /. eigens8]]],
   Max[Re[Flatten[{λ} /. eigens9]]], Max[Re[Flatten[{λ} /. eigens10]]]};
Position[list, _? (# < 0 &)]
```

Out[ ]=
```
{{10}}
```

This principle holds for attachment rate, as well. Notably, if the specialist's infectivity (attachment rate * burst size) is between 1 and 1.9 times that of the generalist, only eigens5 is stable (two bacterial

species with the generalist). If the specialist's infectivity is exactly twice that of the generalist, there is no stable fixed point. If the specialist's infectivity is more than twice that of the generalist, then the only stable fixed point is eigens10 (all four species). Again, this is only true if beta values approximate, rather than exactly equal, 1.

In[ ]:=
```
Clear[mu1, mu2, b1, b2, a1, a2, k1, k2, z1, z2, R, v1, v2, δ]
mu1 = 0.5; mu2 = 0.5;
a1 = 1; a2 = 1;
k1 = 0;
k2 = 0;
R = 2;
δ = 3 / 100; b1 = 0.9999; b2 = 0.9999; v1 = 20;
z1 = 1 / 1000;  z2 = 1.9 / 1000; v2 = 22;
list = {Max[Re[Flatten[{λ} /. eigens1]]],
   Max[Re[Flatten[{λ} /. eigens2]]], Max[Re[Flatten[{λ} /. eigens3]]],
   Max[Re[Flatten[{λ} /. eigens4]]],
   Max[Re[Flatten[{λ} /. eigens5]]], Max[Re[Flatten[{λ} /. eigens6]]],
   Max[Re[Flatten[{λ} /. eigens7]]], Max[Re[Flatten[{λ} /. eigens8]]],
   Max[Re[Flatten[{λ} /. eigens9]]], Max[Re[Flatten[{λ} /. eigens10]]]};
Position[list, _? (# < 0 &)]
```

Out[ ]=
```
{{10}}
```

There are some situations in which the specialist will dominate. Here, *E. coli* has a significant growth disadvantage (low mu) and the specialist has a growth advantage, making the only stable fixed point that of the two bacteria and the specialist.

In[ ]:=
```
Clear[mu1, mu2, b1, b2, a1, a2, k1, k2, z1, z2, R, v1, v2, δ]
mu1 = 0.05; mu2 = 0.5;
a1 = 1; a2 = 1;
k1 = 0;
k2 = 0;
R = 2;
δ = 3 / 100; b1 = 0.9999; b2 = 0.9999;
v1 = 20; z1 = 1 / 1000;  z2 = 1 / 1000; v2 = 60;
list = {Max[Re[Flatten[{λ} /. eigens1]]],
   Max[Re[Flatten[{λ} /. eigens2]]], Max[Re[Flatten[{λ} /. eigens3]]],
   Max[Re[Flatten[{λ} /. eigens4]]],
   Max[Re[Flatten[{λ} /. eigens5]]], Max[Re[Flatten[{λ} /. eigens6]]],
   Max[Re[Flatten[{λ} /. eigens7]]], Max[Re[Flatten[{λ} /. eigens8]]],
   Max[Re[Flatten[{λ} /. eigens9]]], Max[Re[Flatten[{λ} /. eigens10]]]};
Position[list, _? (# < 0 &)]
```

Out[ ]=
```
{{9}}
```

However, when *E. coli* has a significant growth disadvantage imposed through the beta value (b1 > b2)

and the specialist has a growth advantage, then the only stable fixed point is that of all four species.

In[ ]:=
```
Clear[mu1, mu2, b1, b2, a1, a2, k1, k2, z1, z2, R, v1, v2, δ]
mu1 = 0.5; mu2 = 0.5;
a1 = 1; a2 = 1;
k1 = 0;
k2 = 0;
R = 2;
δ = 3 / 100; b1 = 1; b2 = 0.1; v1 = 20; z1 = 1 / 1000; z2 = 1 / 1000; v2 = 60;
list = {Max[Re[Flatten[{λ} /. eigens1]]],
   Max[Re[Flatten[{λ} /. eigens2]]], Max[Re[Flatten[{λ} /. eigens3]]],
   Max[Re[Flatten[{λ} /. eigens4]]],
   Max[Re[Flatten[{λ} /. eigens5]]], Max[Re[Flatten[{λ} /. eigens6]]],
   Max[Re[Flatten[{λ} /. eigens7]]], Max[Re[Flatten[{λ} /. eigens8]]],
   Max[Re[Flatten[{λ} /. eigens9]]], Max[Re[Flatten[{λ} /. eigens10]]]};
Position[list, _?(# < 0 &)]
```

Out[ ]=
```
{{10}}
```
