# Supplementary material for: Bacteriophage specificity is impacted by interactions between bacteria
Source: mSystems. 2024 Feb 20;9(3):e01177-23. doi: 10.1128/msystems.01177-23 (PMC11237722; doi:10.1128/msystems.01177-23)
Supplement: Supplemental material — Supplemental analysis, figures, and tables. [file msystems.01177-23-s0002.docx]

# **Supplemental Materials**

# **Supplemental Analysis**

## Degradation of the generalist phage in minimal media cannot be clearly attributed to any particular media component

We investigated the cause of phage degradation in our minimal media by incubating 10^3^ infectious EH7 particles per well at 37°C with shaking at 432 rotations per minute in glucose minimal media for 48 hours. We found that EH7 degraded below the limit of detection within 24 hours, half the time-period of our standard phage competition assays (Supplemental Figure 2A). However, phage recovery was possible following the addition of cells in some cases (Figure 5B), suggesting that infectious phage particles remained. P22*vir* titer was unchanged over the course of 48 hours (Supplemental Figure 2A; Supplemental Table 5).

Given previous observations that environmental factors such as salinity [1, 2-4], pH [1, 5-7], and metal presence can have significant impacts on phage titer [8-10], we attempted to identify the cause of degradation in our minimal media by removing one component at a time. We created minimal media without metals, without sulfur, without phosphorus, or some combination thereof. Again, we incubated 10^3^ infectious EH7 particles per well at 37°C with shaking at 432 rotations per minute in each minimal media type for 48 hours. However, phage degraded below the limit of detection regardless of which media component was absent, suggesting that metals and osmolarity were not driving the reduction in titer (Supplemental Figure 2B; Supplemental Table 5). Additionally, because phage titer can be reduced as the result of adsorption of viral particles to plastic surfaces, we tested our phage by incubating it in LB [11-12]. We determined that adsorption of phage particles to the plastic was likely not driving the decrease in phage titer, given that EH7 density was unchanged following 48 hours of incubation in LB in the same 96-well plate where degradation in minimal media was observed (Supplemental Figure 2B). This was true despite the low starting phage density, which has been shown to increase the likelihood of rapid phage degradation due to adsorption to plastic [9, 12].

Finally, we investigated which component of LB was responsible for the preservation of phage titer by removing one component at a time. We repeated our previous experiments by incubating 10^3^ infectious EH7 particles per well at 37°C with shaking at 432 rotations per minute in each LB component for 48 hours. The titer of phage incubated in solutions containing tryptone were unchanged over the window of the 48-hour experiment (Supplementary Figure 2B; Supplemental Table 5). When incubated in a solution of yeast extract and salt, the density of EH7 particles was also not significantly changed (Supplementary Figure 2B). However, titer did decrease significantly relative to other conditions when phage were incubated in only yeast or only salt (Supplementary Figure 2B; Supplemental Table 5). These results suggest that tryptone may play a role in the stability of EH7 titer in LB.

# **Supplemental References**

[1] Blazanin M, Lam WT, Vasen E, Chan BK, Turner PE. Decay and Damage of Therapeutic Phage OMKO1 by Environmental Stressors. *PLoS One*. 2022; 17(2): e0263887.

[2] Anderson TF. The reactions of bacterial viruses with their host cells. *The Botanical Review.* 1949; 15 (7):464–505.

[3] Seaman PF, Day MJ. Isolation and characterization of a bacteriophage with an unusually large genome from the Great Salt Plains National Wildlife Refuge, Oklahoma,USA. *FEMS microbiology ecology. 2007*; 60(1):1–13.Epub 2007/01/26.

[4] Whitman PA, Marshall RT. Characterization of two psychrophilic Pseudomonas bacteriophages isolated from ground beef. *Applied microbiology.* 1971; 22(3):463–8.Epub 1971/09/01.

[5] Duyvejonck H, Merabishvili M, Vaneechoutte M, de Soir S, Wright R, Friman VP, Verbeken G, De Vos D, Pirnay JP, Van Melchelen E, Vermeulen SJT. Evaluation of the Stability of Bacteriophages in Different Solutions Suitable for the Production of Magistral Preparations in Belgium. *Viruses*. 2021;13(5): 865.

[6] Jonczyk E, Kłak M, Miedzybrodzki R, Górski A. The influence of external factors on bacteriophages—Review. *Folia Microbiology.* 2011; 56: 191–200.

[7] Molan K, Rahmani R, Krklec D, Brojan M, Stopar D. Phi 6 Bacteriophage Inactivation by Metal Salts, Metal Powders, and Metal Surfaces. *Viruses.* 2022; 14(2): 204.

[8] Yeargin T, Buckley D, Fraser A, Jiang X. The survival and inactivation of enteric viruses on soft surfaces: A systematic review of the literature. *American Journal of Infection Control.* 2016; 44: 1365–1373.

[9] Lin Q, Lim JYC, Xue K, Yew PYM, Owh C, Chee PL, Loh XJ. Sanitizing agents for virus inactivation and disinfection. *View*. 2020; 24: e16.

[10] Richter Ł, Księżarczyk K, Paszkowska K, Janczuk-Richter M, Niedziółka-Jönsson J, Gapiński J, Łoś M, Hołyst R, Paczesny J. Adsorption of Bacteriophages on Polypropylene Labware Affects the Reproducibility of Phage Research. *Scientific Reports*. 2021; 11(1): 7387.

[11] Nakanishi K, Sakiyama T, Imamura K. On the adsorption of proteins on solid surfaces, a common but very complicated phenomenon. *Journal of Bioscience and Bioengineering.* 2011; 91: 233–244.

[12] Rabe M, Verdes D, Seeger S. Understanding protein adsorption phenomena at solid surfaces. *Advances in Colloid and Interface Science.* 2011; 162: 87–106.

# **Supplemental Figures**

**
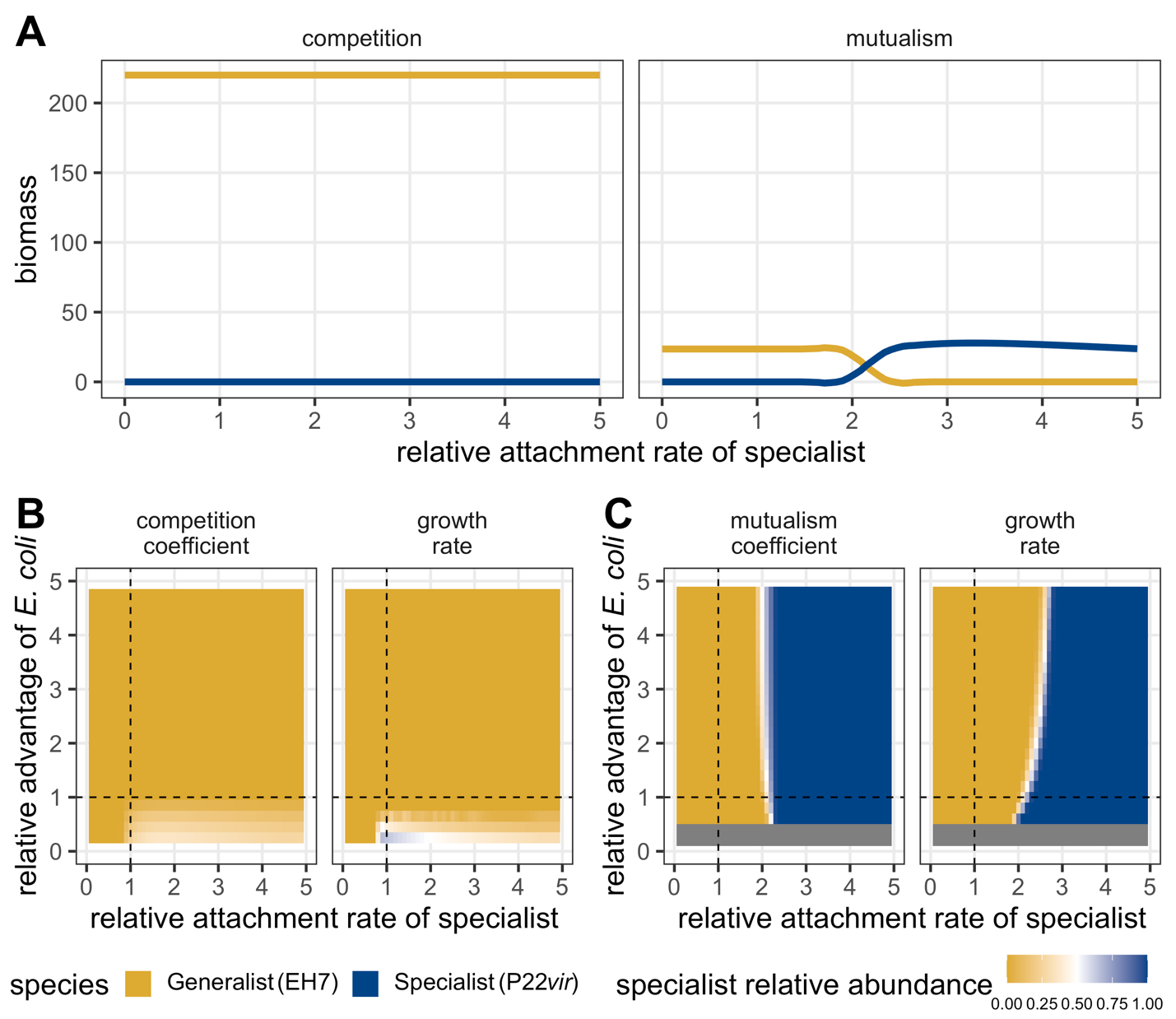
**

**Supplemental Figure 1. End points of numerically-simulated phage dynamics given a variety of parameter trade-offs demonstrate that prey interactions result in different patterns of predator abundance. A:** The final density of each phage type as a function of bacterial interactions and increasing cost of generalism modeled as increasing specialist attachment rate. When prey are mutualistic, an attachment rate above 2.1 favors specialist phage (blue line, P22*vir*) over generalists (yellow line, EH7). When prey compete, there is no relative attachment rate that favors the specialist phage; this is true even as the specialist’s attachment rate increases well beyond the values displayed here. For these analyses, the generalist’s attachment rate is set to 20, with the specialist’s attachment rate increased relative to that value. All other default parameter values can be found in Table 1. **B:** The relative abundance of the specialist phage on competing prey as a function of increasing cost of generalism and relative growth advantage of the alternative prey *E. coli*. Whether prey growth advantage is modeled through growth rate ($\mu$) or competitive coefficients ($\beta$), the generalist is favored (yellow, EH7) except in a small subset of cases where the alternative prey is competitively excluded. For these analyses, the generalist’s attachment rate is set to 1e-3, with the specialist’s attachment rate increased relative to that value. For the competition coefficient panel, the competition coefficient of *S. enterica* is 1, and the competition coefficient of *E. coli* is set relative to that value. For the growth rate panel, the growth rate of *S. enterica* is 0.5, and the growth rate of *E. coli* is set relative to that value. All other default parameter values can be found in Table 1. **C:** The relative abundance of the specialist phage on mutualistic prey as a function of increasing cost of generalism and relative growth advantage of the alternative prey *E. coli*. Whether prey growth advantage is modeled through growth rate ($\mu$) or mutualistic benefit ($\alpha$), a cost of generalism exists above which specialism is favored (blue, P22*vir*). Note that there are benefit and growth rate values for *E. coli* below which the mutualistic system cannot be supported, indicated by the grey bar. For these analyses, the generalist’s attachment rate is set to 1e-3, with the specialist’s attachment rate increased relative to that value. For the mutualism coefficient panel, the mutualism coefficient of *S. enterica* is 1, and the competition coefficient of *E. coli* is set relative to that value. For the growth rate panel, the growth rate of *S. enterica* is 0.5, and the growth rate of *E. coli* is set relative to that value. All other default parameter values can be found in Table 1.

**
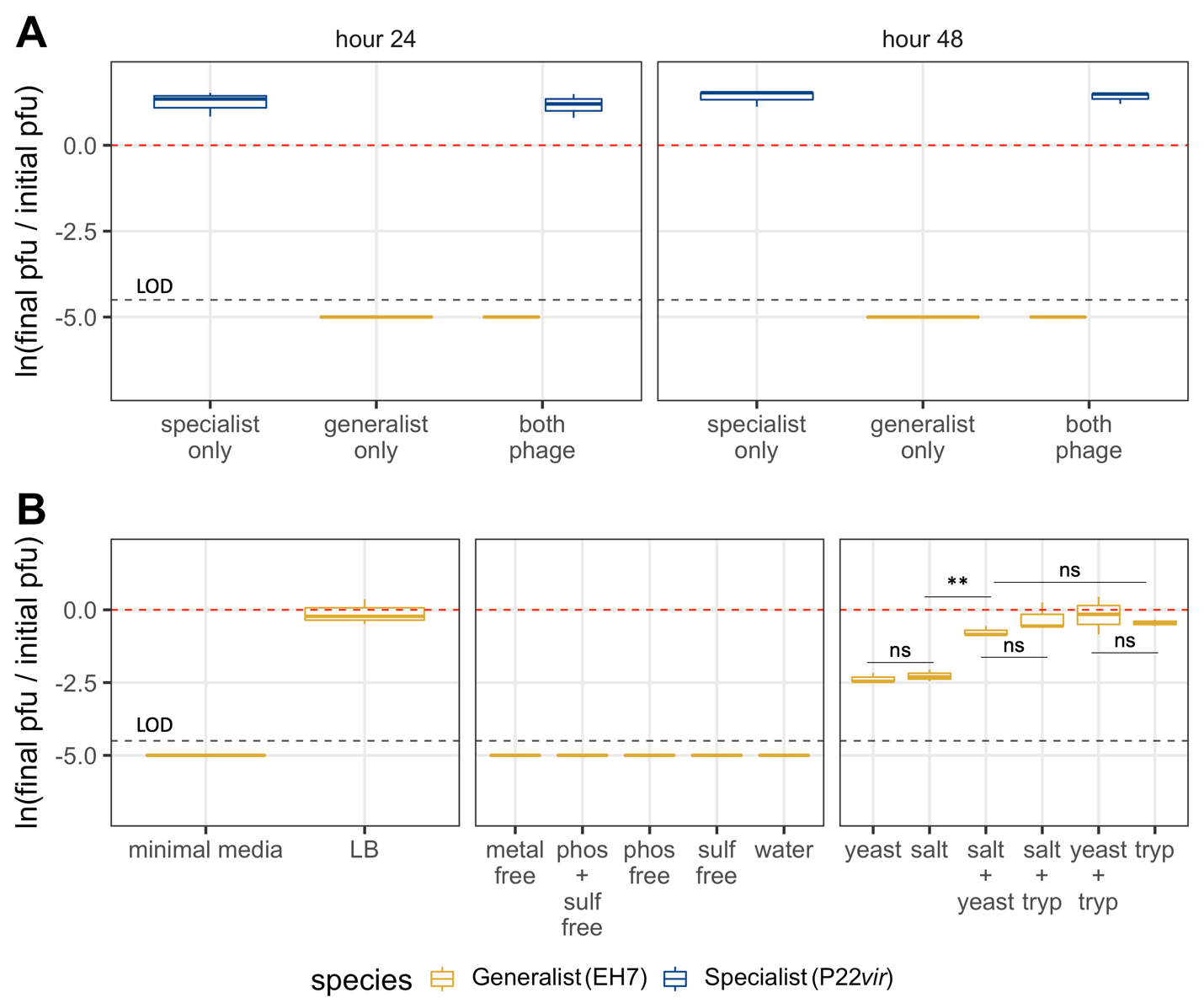
Supplemental Figure 2. Generalist phage degradation differs in minimal media versus LB. A:** EH7 is undetectable after 24 and 48 hours when incubated in minimal media without cells. However, because replication is still possible when cells are added at 24 hours (see Figure 5B, where EH7 titer increases on competitive co-culture even when the addition of cells is delayed), these results suggest that phage are below the limit of detection (LOD) but some infectious particles remain. Statistical significance was determined using a one-way ANOVA with Tukey’s HSD multiple comparison test (Supplemental Table 5). All conditions were incubated in minimal media. **B:** Change in EH7 titer across media conditions. EH7 disappears below the limit of detection in minimal media (p = 0.00035), while in LB without cells, EH7 densities are unchanged over a 48-hour period. Statistical significance for the first facet was determined using a two-tailed t-test (Supplemental Table 5). EH7 degradation is not impacted when different components of the minimal media are removed. Regardless of what component is removed, the phage is undetectable after 48 hours. Change in EH7 titer does differ when different components of LB are removed. Phage is always detectable when at least one component of LB is present, while solutions containing tryptone or both yeast and salt best preserve phage titer over 48 hours of incubation (Supplemental Analysis). Statistical significance for the third facet was determined using a one-way ANOVA with Tukey’s HSD multiple comparison test (Supplemental Table 5). **Note:** in part B, facet panels indicate experiments were completed on different days. **For A and B:** The dotted red line indicates no change in titer from the start of the experiment to the end. Values greater than zero indicate an increase in titer, while values below zero indicate a decrease in titer. The black dotted line indicates the limit of detection (LOD).

# **Supplemental Tables**

**Supplemental Table 1. Morris screening global indices of each ODE parameter on generalist or specialist predator density.** Results of Morris screening analysis, sorted by the impact of each ODE parameter on generalist or specialist predator density at each timestep or time-averaged. Morris screening outputs provide mu, mu* and sigma indices. Global indices are calculated as the square root of mu* squared plus sigma squared. *For Supplemental Table 1: Table is uploaded as an additional file*

**Supplemental Table 2. First order (S) and total effect (T) Sobol’ sensitivity indices for each ODE parameter on generalist or specialist predator density.** Results of Sobol’ sensitivity analysis, sorted by first order and total effect sensitivity indices. Indices indicate the main influence (S) of each ODE parameter or its impact including interactions with other parameters (T) on generalist or specialist predator density at each timestep or time-averaged. *For Supplemental Table 2: Table is uploaded as an additional file*

**Supplemental Table 3. Statistical analysis associated with Figure 4.** Relevant statistical analyses completed for experimental data shown in Figure 4, completed in R v. 4.2.1 using the ggpubr v. 0.4.0 and rstatix v. 0.7.0 packages. *For Supplemental Table 3: Table is uploaded as an additional file*

**Supplemental Table 4. Statistical analysis associated with Figure 5.** Relevant statistical analyses completed for experimental data shown in Figure 5, completed in R v. 4.2.1 using the ggpubr v. 0.4.0 and rstatix v. 0.7.0 packages. *For Supplemental Table 4: Table is uploaded as an additional file*

**Supplemental Table 5. Statistical analysis associated with Supplemental Figure 2.** Relevant statistical analyses completed for experimental data shown in Supplemental Figure 2, completed in R v. 4.2.1 using the ggpubr v. 0.4.0 and rstatix v. 0.7.0 packages. *For Supplemental Table 5: Table is uploaded as an additional file*

| **Parameter** | **Minimum** | **Maximum** |
| --- | --- | --- |
| 𝜶_E,S,_ 𝜶_S,E_ | 0.1 | 2.5 |
| 𝜷_E,S,_ 𝜷_S,E_ | 0.1 | 2.5 |
| 𝝁_E,_ 𝝁_S_ | 0.1 | 2.5 |
| 𝜸_E,G_, 𝜸_S,G,_ 𝜸_S,P_ | 15 | 65 |
| 𝜻_E,G,_ 𝜻_S,G,_ 𝜻_S,P_ | 0.0009 | 0.01 |
| 𝛅_E,_ 𝛅_S,_ 𝛅_G,_ 𝛅_P_ | 0.0009 | 0.1 |
| 𝞳_E,_ 𝞳_S_ | 0.1 | 10 |
| ***R*** | 0 | 5 |

**Supplemental Table 6. Sobol’ sensitivity analysis and Morris screening parameter ranges.** Minimum and maximum uniform distribution values used for each ODE parameter in Morris screening and Sobol’ sensitivity analyses.

| **Strain ancestor** | **Antibiotic marker** | **Fluorescent marker** | **Source** | **Relevant phenotype** |
| --- | --- | --- | --- | --- |
| *S. enterica* LT2 *metA** *metJ** | kanR | YFP | Harcombe 2010 | Methionine hypersecreter |
| *E. coli* K-12 BW25113 ∆*metB* | kanR | CFP | Harcombe 2010 | Methionine auxotroph |
| *E. coli* K-12 BW25113 ∆*trxA* | kanR | NA | Baba et al. 2006 | Sensitive to EH7, resistant to P22*vir* |
| *S. enterica* serovar Typhimurium NCTC 74 ∆*btuB* | NA | NA | S. Bowden | Sensitive to P22*vir*, resistant to EH7 |
| P22*vir* | NA | NA | I. J. Molineaux | *S. enterica*-specific phage |
| EH7 | NA | NA | E. Hansen | Generalist phage |

**Supplemental Table 7. Phage and bacterial strains.** Strains used for experiments. See materials and methods for additional details.

| **Predicted mutations** | | | | | |
| --- | --- | --- | --- | --- | --- |
| **position** | **mutation** | **freq** | **annotation** | **gene** | **description** |
| 30,899 | G→T | 100% | intergenic (-240/+114) | 24 ← / ← c2 | unknown/prophage repressor |
| 31,700 | C→G | 100% | intergenic (-37/-44) | c2 ← / → cro | prophage repressor/repressor |
| 31,716 | A→G | 100% | intergenic (-53/-28) | c2 ← / → cro | prophage repressor/repressor |

**Supplemental Table 8. *breseq* predictions of point mutations responsible for repressing lysogeny in P22*vir.*** *breseq* predictions of point mutations in P22*vir* strain used for all experiments relative to the ancestral, lysogenic version of P22 (GenBank accession NC_002371.2). Sequencing of the lab phage strain was completed by Seq Center, LLC (<https://www.seqcenter.com/>).

| **Type** | **Component** | **Metric** | **S mono** | **E mono** | **ES mutualism** | **ES competition** |
| --- | --- | --- | --- | --- | --- | --- |
| C source | lactose | mM | 0 | 0 | 2.9 | 0 |
|  | glucose | mM | 5.6 | 5.6 | 0 | 5.6 |
| auxotroph amendments | methionine | mM | 0 | 0.5 | 0 | 0.5 |
| SO4 source | (NH4)2SO4 | mM | 3.7 | 3.7 | 3.7 | 3.7 |
|  | MgSO4 | mM | 0.814 | 0.814 | 0.814 | 0.814 |
| P source | K2HPO4 | mM | 14.5 | 14.5 | 14.5 | 14.5 |
|  | NaH2PO4 | mM | 16.3 | 16.3 | 16.3 | 16.3 |
| Metals | ZnSO4 | µM | 1.2 | 1.2 | 1.2 | 1.2 |
|  | MnCl2 | µM | 1 | 1 | 1 | 1 |
|  | FeSO4 | µM | 18 | 18 | 18 | 18 |
|  | (NH4)6Mo7O24 | µM | 2 | 2 | 2 | 2 |
|  | CuSO4 | µM | 1 | 1 | 1 | 1 |
|  | CoCl2 | µM | 2 | 2 | 2 | 2 |
|  | Na2WO4 | µM | 0.33 | 0.33 | 0.33 | 0.33 |
|  | CaCl2 | µM | 20 | 20 | 20 | 20 |

**Supplemental Table 9. Hypho minimal media composition.** Media composition for mutualistic and competitive communities, as well as bacterial monocultures. Note that phage degradation assays on starved cells were completed in ES mutualism media.

# **Supplemental Files**

**Supplemental File 1. PDF of Mathematica notebook for fixed point stability analysis.** Fixed point stability analyses completed in Mathematica 13.2.1. *For Supplemental File 1: PDF is uploaded as an additional file.*
